# Ultra-high static magnetic field induces a change in the spectrum but not frequency of DNA spontaneous mutations in *Arabidopsis thaliana*

**DOI:** 10.3389/fpls.2023.1305069

**Published:** 2023-12-06

**Authors:** Xiang Xu, Mengjiao Chen, Tianli Chen, Xinda Ni, Zhicai Fang, Yanwen Fang, Lei Zhang, Xin Zhang, Jirong Huang

**Affiliations:** ^1^ Shanghai Key Laboratory of Plant Molecular Sciences, College of Life Sciences, Shanghai Normal University, Shanghai, China; ^2^ Heye Health Industrial Research Institute of Heye Health Technology Co., Ltd., Huzhou, China; ^3^ High Magnetic Field Laboratory, Key Laboratory of High Magnetic Field and Ion Beam Physical Biology, Hefei Institutes of Physical Science, Chinese Academy of Sciences, Hefei, China

**Keywords:** magnetic field, mutagenicity, DNA, mutation, genome, *Arabidopsis*

## Abstract

Biological effects of magnetic fields have been extensively studied in plants, microorganisms and animals, and applications of magnetic fields in regulation of plant growth and phytoprotection is a promising field in sustainable agriculture. However, the effect of magnetic fields especially ultra-high static magnetic field (UHSMF) on genomic stability is largely unclear. Here, we investigated the mutagenicity of 24.5, 30.5 and 33.0 T UHSMFs with the gradient of 150, 95 and 0 T/m, respectively, via whole genome sequencing. Our results showed that 1 h exposure of *Arabidopsis* dried seeds to UHSMFs has no significant effect on the average rate of DNA mutations including single nucleotide variations and InDels (insertions and deletions) in comparison with the control, but 33.0 T and 24.5 T treatments lead to a significant change in the rate of nucleotide transitions and InDels longer than 3 bp, respectively, suggesting that both strength and gradient of UHSMF impact molecular spectrum of DNA mutations. We also found that the decreased transition rate in UHSMF groups is correlated with the upstream flanking sequences of G and C mutation sites. Furthermore, the germination rate of seeds exposed to 24.5 T SMF with -150 T/m gradient showed a significant decrease at 24 hours after sowing. Overall, our data lay a basis for precisely assessing the potential risk of UHSMF on DNA stability, and for elucidating molecular mechanism underlying gradient SMF-regulated biological processes in the future.

## Introduction

Magnetic fields have been widely utilized in the basic research of life sciences as well as in various facilities including nuclear magnetic resonance (NMR) and magnetic resonance imaging (MRI) due to the non-invasive advantage. The effects of magnetic fields on plant biological processes, such as seed germination, root growth, flowering, photosynthesis and stress resistance have been wildly reported (Maffei, 2014; Radhakrishnan, 2019; Tapia-Belmonte et al., 2023). Based on the magnetic field effects, developing of smart facilities for plant growth control and phytoprotection will be a promising solution in the development of sustainable agriculture in the future.

Magnetic biological effects are closely related to the properties of magnetic fields, which are primarily classified into the two distinctive types, static magnetic field and alternating/electronic magnetic field (EMF), depending on whether magnetic density and/or direction are constant or fluctuant over time. In the field of magnetic bioeffect research, static magnetic field (SMF) is further divided into four subtypes: weak (< 1 mT), moderate (1 mT to 1 T), strong (1 to 5 T) and ultra-high (> 5 T) fields (Dini and Abbro, 2005), whereas non ionizing EMF is classified into extremely low frequency (ELE), intermediate frequency (IF) and radiofrequency (RF) fields (Hartwig et al., 2009). Considering the increasing availability of high and ultra-high SMF, SMF with stronger intensity will be studied and utilized for their possible higher biological effects. To prevent the potential hazards caused by magnetic fields, International Commission on Non Ionizing Radiation Protection (ICNIRP) has recommended 400 mT static magnetic field (SMF) as a safe limit for general public exposure of any part of the body, while 8 T for the occupational exposure of the limbs (Hartwig et al., 2009). In contrast, the hazardous effects of SMF on plant growth and development have been ignored. In addition, the impacts of inevitable magnetic pollution, especially for ultra-high magnetic field, on environment and living organisms like insects are largely unknown. For example, whether UHSMF exposure leads to irreversible genetic damages has remained uncertain.

At the DNA macromolecule level, a consensus behavior was reported that cellular DNA chains and chromosomes aligned or moved perpendicularly to the magnetic field orientation in UHSMF due to the diamagnetic anisotropy of nucleic acid bases (Maret et al., 1975; Zhang et al., 2017a). However, published data about UHSMF-induced DNA damages were not consistent. Nakahara et al. (2002) showed no difference in the frequency of micronucleus formation, which is caused by the failure of repair of DNA breaks, between 4-day 10 T-treated and -untreated Chinese hamster ovary K1 cells (Nakahara et al., 2002). In *E. Coli*, mutagenic effects of UHSMF were not found in the wild-type strain, but was observed in mutant strains with defects in DNA repair, suggesting that UHSMF is able to induce DNA mutations indirectly (Zhang et al., 2003). There were eleven published reports that evaluated effects of clinical magnetic resonance imaging exposures on DNA stability with the human blood samples. Among them, six studies showed an increase while five showed no difference in the number of DNA double-strand breaks (DSB) or micronuclei (Vijayalaxmi et al., 2015). With the rapid development of both genome sequencing and superconductive materials, it becomes possible to systematically and quantitatively evaluate genotoxicity of UHSMF. Here, we used dried seeds of the model plant *Arabidopsis thaliana* as the material to assess genotoxic effects of UHSMF up to 33.0 T via genome sequencing. Our results showed that UHSMF exposures from 24.5 to 33.0 T do not enhance the rate but alter spectra of DNA mutations in the descendants of *Arabidopsis*.

## Results

### Analysis and verification of genome sequencing data

**Figure 1:**
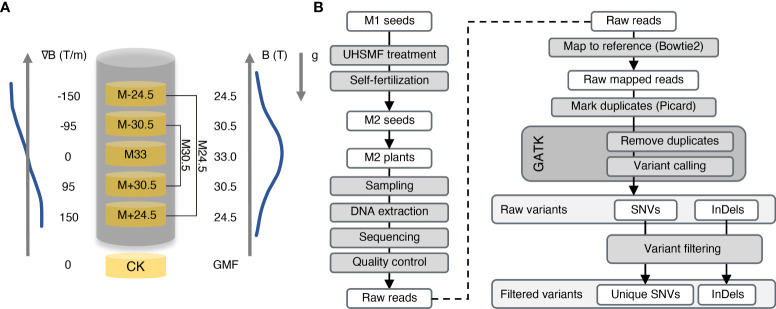
Overview of UHSMF treatment, sample preparation and data analyses. **(A)** Parameters of UHSMF and UHSMF treatments including M24.5 (M-24.5 and M+24.5), M33, and M30.5 (M-30.5 and M+30.5). The seeds without UHSMF treatment, namely GMF (geomagnetic field), were used as a control (CK). B (T), magnetic flux density; ∇B (T/m), magnetic gradient; g, gravity; arrowheads, directions of parameters. **(B)** Flow diagram of sample preparation and data analyses. UHSMF-treated dried seeds (M1 seeds) were self-fertilized for one generation (M2 seeds), and M2 plants were sampled for DNA extraction and genomic sequencing. Preliminary analysis was performed through variants calling and selecting. GATK, GenomeAnalysisTK; SNV, single nucleotide variants; InDel, insertion and deletion.

To evaluate the effect of UHSMF on genotoxicity, we exposed *Arabidopsis* dried seeds to three strengths of UHSMF for 1 h. UHSMF was generated by a home-built water-cooled magnet at the National Major Scientific and Technological Infrastructure located in Hefei, China (Gao et al., 2016; Tian et al., 2021). The three treatments were designated as M33, M30.5 and M24.5, in which the number represents magnetic strength in Tesla (T), with a gradient of 0, 95 and 150 T/m, respectively (**Figure 1A**). The seeds without UHSMF treatment, namely geomagnetic filed (GMF), were used as a control (CK). Since the highest strength of SMF was produced in the center of the magnet, the orientation of the magnetic field gradient bellow the center was the same as that of the gravity, which mimics the hypergravity condition, while that above the center mimics the hypogravity condition. Thus, M30.5 and M24.5 treatments included hypergravity (M+30.5 and M+24.4) and hypogravity (M-30.5 and M-24.5) conditions.

**Figure 2:**
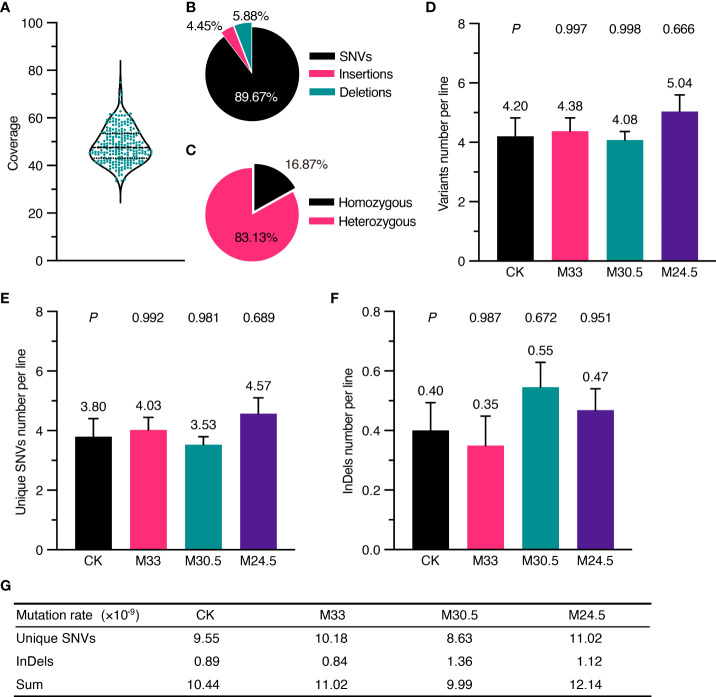
Depth of whole genomic sequencing and UHSMF effect on DNA mutations including SNV and InDel. **(A)** Coverage of sequencing depth for all the samples. **(B)** Percentage of three types of mutations. **(C)** Percentage of homozygous and heterozygous mutations. **(D-F)** Average number of filtered variants **(D)** including unique SNVs **(E)** and InDels **(F)**. The bars indicate standard error. Figures above error bars mean the number of mutations per line. *P* values were calculated using one-way ANOVA Tukey`s multiple comparison test between UHSMF and CK samples. **(G)** Frequency of unique SNVs and InDels per site per line per generation.

The *Arabidopsis* seeds used in the study were the Col-0 ecotype, whose genome was completely sequenced with high quality in 2000 (Kaul et al., 2000). To identify inheritable mutations possibly induced by UHSMF, we propagated the UHSMF-treated and CK seeds for one generation by self-fertilization, and harvested the seeds as individual plants. In each treatment, a total of 40 independent siblings were randomly selected from the population of the second generation for DNA extraction (**Figure 1B**). Each DNA sample was sequenced by Illumina next-generation technology to reach a coverage depth of 50 folds. Ultimately, we obtained 236 individual genomic sequences with coverage from 33.4 to 74.9 depth (**Figure 2A**). The obtained raw reads were first analyzed and mapped to the reference genome (TAIR10, www.arabidopsis.org) with Bowtie2 program (Langmead and Salzberg, 2012). Sequence variants including single nucleotide variants (SNVs) and InDels (insertions and deletions) ranged from 1 to 28 bp were called by Genome Analysis Toolkit (McKenna et al., 2010) (GATK) (**Figure 1B**). On average, each line contained ~1,600 variants (**Supplementary Figure 1A**). Since this number is much higher than expected (Ossowski et al., 2010), we inferred that it could be due to many variants already present in the seeds before UHSMF treatment, i.e. germline mutations. To solve this problem, we assumed that it is an extremely low possibility that new mutations took place at the same nucleotide position of the genome. Thus, we retained the unique variants (i.e. singletons) in each sample and removed those overlapped with any other ones in all samples. In total, we identified 1,055 singleton variants including 946 SNVs (89.67%) and 109 InDels that consisted of 47 insertions (4.45%) and 62 deletions (5.88%) (**Supplementary Table 1**, **Figure 2B**). Among these variants, 178 sites (16.87%) were homozygous while the others (83.13%) were heterozygous (**Figure 2C**). In addition, most (77.06%) of the InDels were short, with the size of 1-3 bp (**Supplementary Figure 2**). Generally, our data are in agreement with previous reports that SNVs prevailed over InDels (Ossowski et al., 2010; Jiang et al., 2011; Lee et al., 2012). To verify these calls, we randomly selected 56 SNVs and 23 InDels, which were checked by PCR-based Sanger sequencing. Our results showed that 54 (96.43%) SNVs and 22 (95.65%) InDels were confirmed (**Supplementary Figure 3**), indicating that the accuracy of the variants called by our analytic approaches are compelling.

### UHSMF exposure of dried seeds for 1 h has no effect on the rate of heritable spontaneous mutations

To quantify the effect of UHSMF on DNA stability, we calculated the average number of mutations per line for each treatment. In general, the orientation of magnetic force, which is generated by magnetic gradient, is ignored in the study of magnetic bioeffects. Here, we first examined the effect of two orientations, namely hypergravity and hypogravity, with the identical strength and gradient on DNA mutations. Our data showed that M+24.5 and M-24.5 or M+30.5 and M-30.5 treatments did not significantly influence the average number of DNA mutations including SNVs and InDels (**Supplementary Figures 1B-D, 4A-C**), indicating that differential orientations with either 95 or 150 T/m have no impact on DNA stability. To focus our analysis on DNA mutation affected by magnetic strength and gradient, we combined the two sets of data from M+24.5 and M-24.5 into one (M24.5), and from M+30.5 and M-30.5 into M30.5. In addition, we confirmed that both the transition rate and the number of InDels larger than 3 bp, which were influenced by UHSMF (discussed latter in this study), were not significantly changed between M+30.5 and M-30.5 or between M+24.5 and M-24.5 (**Supplementary Figures 4D, E**).

We estimated the number of the overall mutations including SNV and InDel per line in each treatment. The highest number of variants per line were found in M24.5 treatment (5.04), followed by M33 (4.38), CK (4.20), and M30.5 (4.08) (**Figure 2D**). However, the differences between any UHSMF treatment and CK were not statistically significant (one-way ANOVA, *P* > 0.05). We then analyzed the SMF effect on SNV and InDel independently. The number of SNVs per line were the lowest in M30.5 (3.53) and the highest in M24.5 (4.57), while average InDels ranged from 0.35 (M33) to 0.55 (M30.5). M24.5 had the highest SNVs number (4.57) while M33 contained the lowest InDel (0.35). However, no statistically significant differences of both SNVs and InDels were detected between UHSMF-exposed seeds and CK (one-way ANOVA, *P* > 0.05) (**Figures 2E, F**). To estimate the average mutation rates of SNV and InDel per generation per site per line, we used the sum of homozygous mutations and half of the total heterozygous mutations due to the 50% probability for a heterozygote to be segregated into a homozygote in the offspring. Our data showed that the average mutation rates of SNV and InDel in CK were 9.55 and 0.89 × 10^-9^, respectively, which had no statistical difference from the overall UHSMF treated samples (one-way ANOVA, *P* > 0.05) (**Figure 2G**). Likewise, the average rates of the total mutations (SNV and InDel) in UHSMF-treated samples were similar to those in CK (**Figure 2G**). Taken together, our data suggest that 1 h exposure of UHSMF with the intensity from 24.5 to 33 T has no impact on the rate of heritable DNA mutations.

### UHSMF exposure leads to a decrease in the spectrum of SNVs

**Figure 3:**
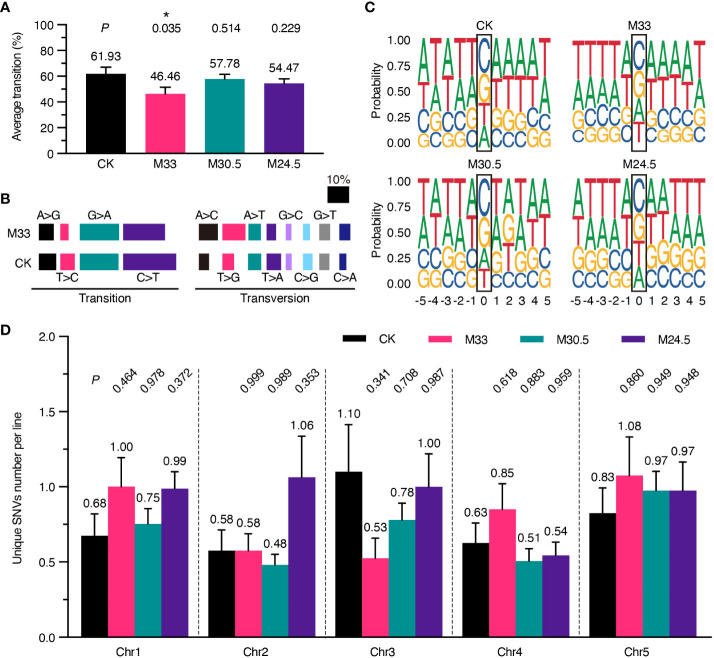
Analysis of the spectrum of DNA mutations, consensus of flanking sequences and chromosomal distribution of mutations. **(A)** Percentage of transition per line. *P* values (*t* test) between UHSMF and CK samples were shown on the top of each treatment. **(B)** Percentage of base substitutions including transition and transversion in CK and M33. Each type of base substitution was shown in different colors. **(C)** Consensus analysis of the five nucleotides up- and down-stream flanking the unique SNVs. The height of base letters indicates their proportion in the corresponding site. The unique SNV sites are marked by black frame. **(D)** Chromosomal distribution of unique SNVs per line. All tests showed no statistical difference (*P* > 0.05, one-way ANOVA Tukey`s multiple comparison test). Asterisk means *P* < 0.05.

To analyze possible genotoxic effects of UHSMF in more detail, we examined whether the spectrum of SNVs is altered in the UHSMF-treated groups in comparison with the CK. Consistent with the published data about the bias of SNVs in *A. thaliana* (Ossowski et al., 2010), *E. coli* (Lee et al., 2012), *D. melanogaster* (Keightley et al., 2009) and *H. sapiens* (Lynch, 2010), the rate of transitions (purine-to-purine or pyrimidine-to-pyrimidine changes) was higher than that of transversions (interchanges between purine and pyrimidine), and the two transitions of C-to-T and G-to-A were most prevalent among all single nucleotide substitutions in CK (**Figures 3A, B**). Although the rate of nucleotide transitions was lower in all UHSMF-treated groups in comparison with CK, a significantly low rate of transitions was detected only in M33 treatment (**Figure 3A**). Further analysis indicated that the most reduced transitions in M33 were the pyrimidine-to-pyrimidine substitution (32.24% to 24.22%), whereas the most increased transversions were A-to-C (5.26% to 9.32%) and T-to-G (5.92% to 11.18%) (**Figure 3B**). Taken together, our data suggest that M33 treatment leads to a significant decrease in the transition rate, particularly in pyrimidine-to-pyrimidine mutations.

Since the C-to-T and G-to-A transitions are predominant in all SNVs, we assumed that the induction or repair of mutations under UHSMF conditions may have a bias on the base type or motif flanking at the mutational site. To test this hypothesis, we analyzed the consensus of the 5 nucleotides flanking the left and right side of all the SNV sites, and visualized by ggseqlogo (version 0.1) (Wagih, 2017). As shown in **Figure 3C**, the three UHSMF groups displayed the same order as CK with C as the top and G as the second in the mutation frequency. However, the third and fourth mutation frequency were T and A, respectively, in CK and M24.5, and were A and T, respectively, in M33 and M30.5. These results indicate that UHSMF has minor effect on the order of nucleotide mutation frequency. Interestingly, we found that the consensus at the upstream -3 to -1 position flanking the C site was TTA in UHSMF groups but ATT in CK, while flanking the G site was AAT in UHSMF groups but TAA in CK (**Figure 3C**). No consensus of nucleotides was observed at the -5, and -4 positions and at the downstream of the mutation C and G sites in all UHSMF groups. In addition, we analyzed the flanking sequences of the mutated nucleotides, and did not find any typical motifs shared by UHSMF groups, compared to CK (**Supplementary Figure 5**). These results suggest that the reduced rate of transitions in UHSMF is somewhat related to the characteristic of the upstream flanking sequences of the SNVs.

We then analyzed whether UHSMF has an impact on the distribution of SNVs on the five *Arabidopsis* chromosomes (Chr). The number of SNVs estimated in each chromosome per line ranged from the lowest of 0.48 (Chr2 in M30.5) to the highest of 1.1 (Chr3 in CK) (**Figure 3D**). We did not detect significant differences in the average number of SNVs distributed on each Chr between UHSMF and CK (**Figure 3D**). These results indicate that the Chr distribution of the spontaneous mutations for SNVs is not altered by UHSMF treatment.

### M24.5 treatment leads to an increase in the number of InDels larger than 3 bp

**Figure 4:**
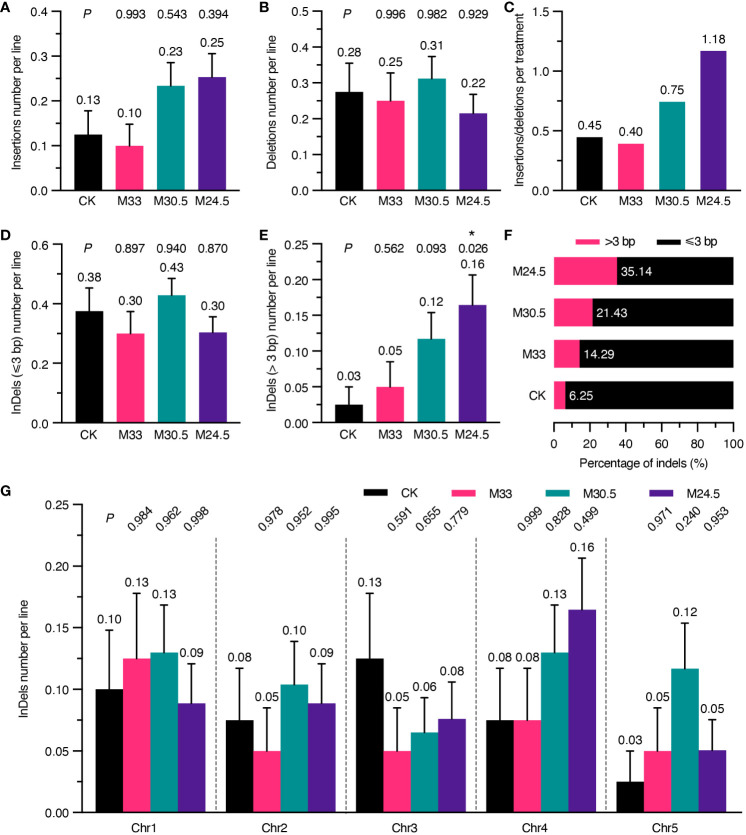
Analysis of UHSMF effect on the components and chromosomal distribution of InDels. **(A, B)** Comparison of the number of insertions **(A)** and deletions **(B)** per line between CK and UHSMF exposed groups. **(C)** The ratio of insertions to deletions in CK and UHSMF-treated groups. **(D, E)** Comparison of InDels with a length less than or equal to 3 **(D)** and larger than 3 **(E)** between CK and UHSMF-exposed groups. **(F)** Percentage of long (> 3) and short (≤ 3) InDels. **(G)** Chromosomal distribution of the number of InDels per line in CK and UHSMF-exposed groups. *P* values between CK and UHSMF treatments in **(A, B, D)**, and **(G)** were calculated via one-way ANOVA Tukey`s multiple comparison test, and in e was calculated via student’s *t* test. Error bars indicate standard error. The average number of InDels are shown above error bars. Asterisk means *P* < 0.05.

Considering that UHSMF exposure can change the spectrum of SNVs, we assumed that it also affects the spectrum of InDels. Therefore, we analyzed the number of InDels per line independently. As shown in **Figure 4A**, the number of insertions in M30.5 and M24.5 were 0.23 and 0.25 per line, respectively, which were about two-fold of, but not significantly different from those in CK and M33. In contrast, the number of deletions were similar among all groups (**Figure 4B**). Analysis of the ratio between insertions and deletions clearly showed that M24.5 (1.18) and M30.5 (0.75) were much higher than CK (0.45) and M33 (0.4) (**Figure 4C**). These data suggest that the ratio of insertions to deletions is positively correlated with the gradient in spite of no significant effect of UHSMF on the number of insertions.

We then dissected the effect of UHSMF on the size of InDels. Our data showed that the number of InDels of 1 to 3 bp per line were similar between UHSMF treatments and CK (**Figure 4D**). However, there were obvious differences in InDels larger than 3 bp among the four groups. The number of such large InDels per line was 0.16 in M24.5, significantly higher than that in CK (0.03) (**Figure 4E**), and the percentage of the large InDels gradually increased with an increase in the magnetic gradient (**Figure 4F**). We did not observe any significant differences in distribution of InDels across the five Chrs (**Figure 4G**). In addition, we also analyzed the flanking sequences of InDels, and found that approximately 50% of the InDels were adjacent to a homopolymer or polynucleotide repeat in each group (**Supplementary Figure 6A**, **Supplementary Table 2**). InDels smaller than 3 bp showed a similar pattern in CK and UHSMF treatments while homopolymeric and polynucleotide repeat InDels with larger than 3 bp were only present in M30.5 and M24.5, respectively (**Supplementary Figures 6B, C**). Meanwhile, flanking sequence with homopolymers was more frequently detected for insertions but less for deletions in CK and M24.5 (**Supplementary Figures 6D, E**). Taken together, our results suggest that UHSMF gradient is positively correlated with the number of InDels larger than 3 bp.

### UHSMF exposure does not alter genome-wide distribution and deleteriousness of spontaneous mutations

We mapped all mutations in the genome-wide scale, and found that mutations were randomly distributed across five chromosomes in all groups, except that a relatively greater density of mutations was observed in the proximity of the centromere, particularly on Chr3 (**Supplementary Figure 7**). In fact, such a distribution pattern of mutations was already reported in *Arabidopsis* (Ossowski et al., 2010). These results suggest that UHSMF exposure has no bias effect on mutation distribution across chromosomes.

**Figure 5:**
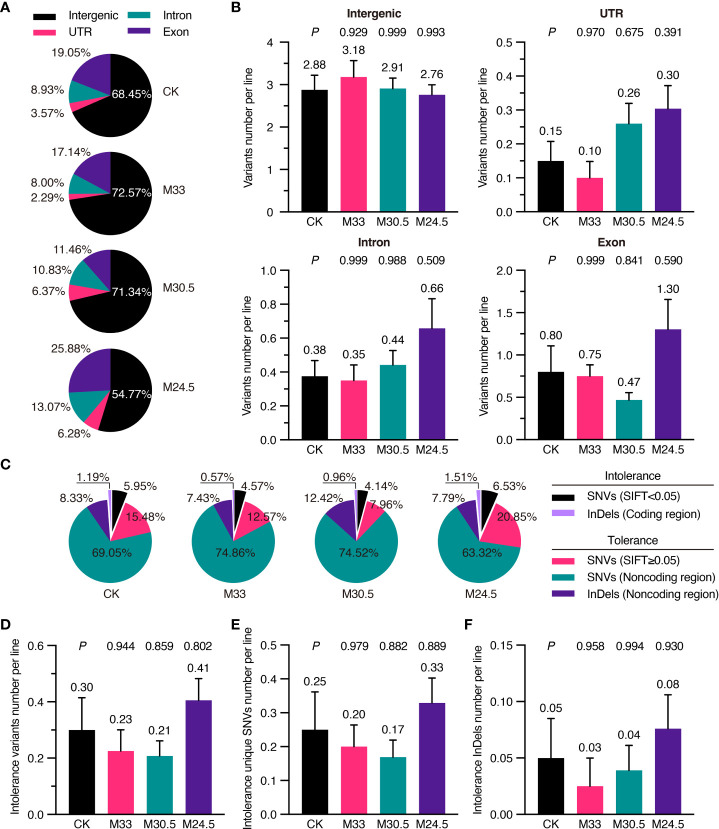
Functional classification and SIFT analysis of mutations in UHSMF and CK groups. **(A)** Percentage of intergenic, UTR (untranslated region), exonic and intronic mutations in UHSMF and CK groups. **(B)** The number of the four mutations per line in UHSMF and CK groups. **(C)** SIFT analysis of the variants in the five individual groups. SNVs with SIFT score less than 0.05 and InDels in the coding sequences are likely harm to protein functions and are named as intolerance variants. **(D-F)** The number of intolerance variants per line **(D)** including unique SNVs **(E)** and InDels **(F)**. In **(B)** and **(D-F)**, the average number of each group was shown above the error bar. *P* value between UHSMF and CK groups was computed via one-way ANOVA Tukey`s multiple comparison test. Error bars indicate standard error of the mean.

To predict whether mutations affect gene function and expression, we analyzed deleteriousness of all SNVs depending on their sites in intergenic region, untranslated region (UTR), intron and exon based on TAIR10 annotation (Cingolani et al., 2012). In general, most (from 54.77 to 72.57%) of the variants were located in the intergenic region, followed by in the exon (11.46 to 25.88%), intron (8.00 to 13.07%) and UTR (2.29 to 6.28%) in all groups. Interestingly, M24.5 treatment with the largest gradient resulted in the highest mutation (25.88%) in exons while the lowest mutation (54.77%) in intergenic regions (**Figure 5A**). However, no significant difference was detected in the average number of variants in exons and other regions among all the groups (**Figure 5B**).

To further check the potential effects of exon mutations, we analyzed variants deleteriousness via the Sorting Intolerant From Tolerant (SIFT) algorithm, namely SIFT4G (Vaser et al., 2016). SIFT4G predicted deleterious effects ranging from 0 (deleterious) to 1 (tolerated) on amino acid changes. It is generally recognized that the variants with score less than 0.05 are deleterious. Based on this criterion, 7.14% of the variants in CK were deleterious (or intolerance), including the deleterious SNVs (SIFT < 0.05) and InDels in coding region (**Figure 5C**). Compared to that of CK (7.14%), proportion of deleterious variants was higher in M24.5 (8.04%) while lower in M33 (5.14%) and M30.5 (5.10%). On average, the number of deleterious variants were ranged from 0.21 to 0.41 per line in CK and UHSMF groups, and no significant difference was detected between CK and UHSMF samples (**Figure 5D**). Likewise, there were no significant differences in the number of deleterious SNVs and InDels between UHSMF treatments and CK (**Figures 5E, F**). Taken together, our data indicate that UHSMF exposure has no effects on genome-wide distribution and deleteriousness of spontaneous mutations.

### UHSMF exposure affects seed germination at the early stage

**Figure 6:**
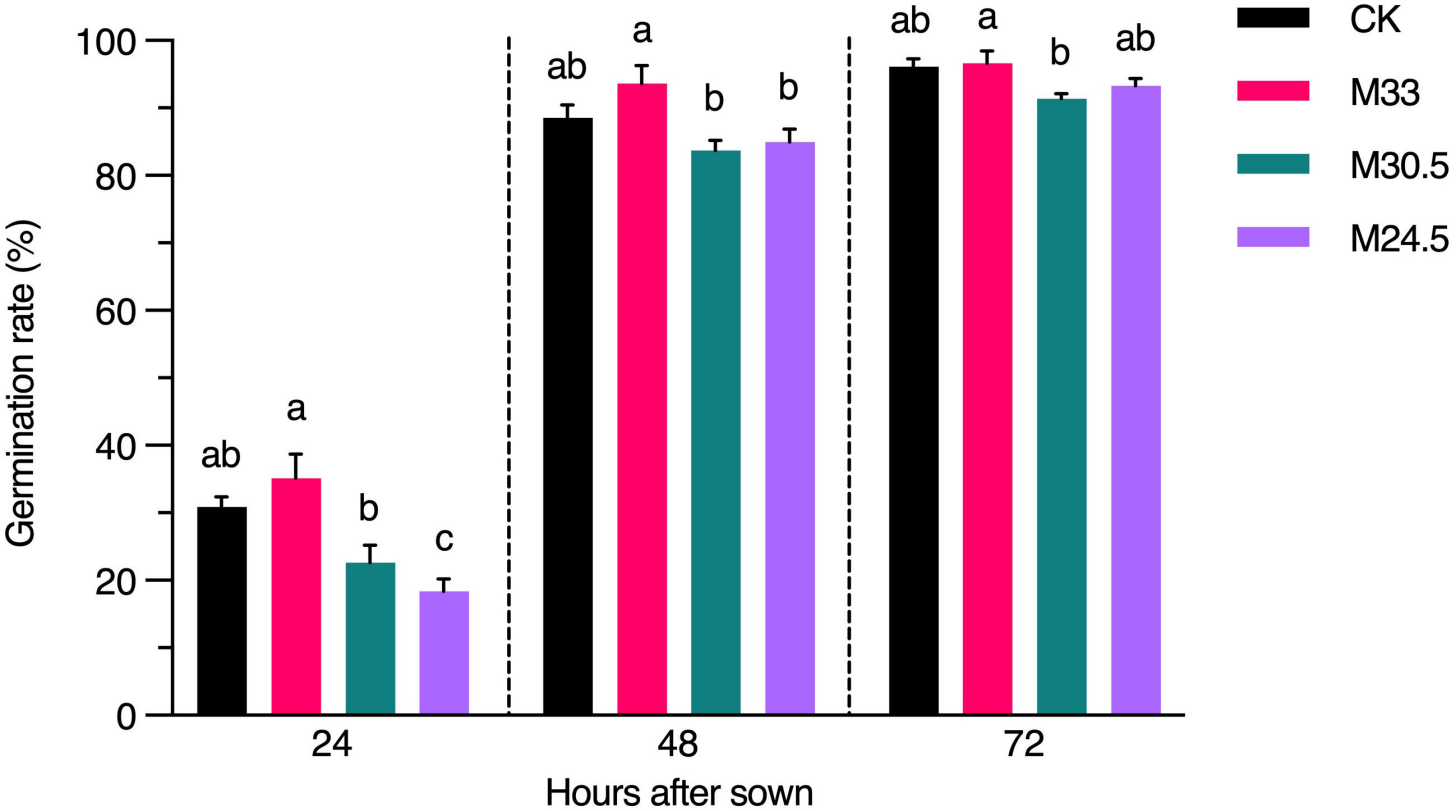
Effect of UHSMF on seed germination rate. The average germination rate of seeds in light (100 μmol·m^-2^·s^-1^) at 20°C. The data were shown as means ± s.e. (n ≥ 40). Statistical difference (one way ANOVA, *P* < 0.05) was marked with different lower-case letters.

To investigate whether UHSMF exposure has a direct effect on seed physiology, we inspected the rate of seed germination in a time course. Our data showed that except for a significant decrease in germination rate of M24.5-expoured seeds at 24 h after seed sowing, other UHSMF treatments had no significant effect on germination rate examined at 24, 48 and 72 h, although a general tendency was observed that germination was faster for M33 seeds but slower for M30.5 and M24.5 seeds, compared to CK (**Figure 6**). In addition, we found that among the three groups of SMF-exposed seeds, M33 seeds maintained significantly higher germination rate than M24.5 and M30 during the whole germination process, except for M24.5 at 72 h after sowing, while no significant difference in germination rate between M24.5 and M30.5 seeds except for at 24 h after sowing. These results suggest that the gradient but not the strength of SMF has a negative effect on seed germination, particularly at the early stage.

To check the potential impact of genetic variations on seed germination especially in M24.5, genes responsible for the mutation sites were annotated with Gene Ontology (GO) biological process by Metascape (Zhou et al., 2019). Throughout the annotation results, we only found the *HEAT SHOCK COGNATE PROTEIN 70-1* (*HSC70-1*, AT5G02500), which mutated in M24.5 with SIFT score 0, was involved in seed germination process (**Supplementary Table 3**). However, the function of HSC70-1 was redundant with HSC70-2 and HSC70-3 (Zhao et al., 2021). Thus, the germination rate changes may be not due to variants in lines of *HSC70-1*. It is not clear whether epigenetic variants occurred in these lines and subsequently affect the germination rates.

## Discussion

In this study, we evaluated the effects of 1 h exposure of the three UHSMF (33 T, 0 T/m; 30.5 T, 95 T/m; and 24.5 T, 150 T/m) on seed germination and genetic mutation in *Arabidopsis thaliana*. In general, our data showed that the bioeffects of UHSMF relies not only on the strength but also on the gradient. In physiology, the uniform M33 promoted seed germination while the gradient M30.5 and M24.5 inhibited seed germination, particularly at the early stage (24 h after sowing). In genetics, although UHSMF had no significant effects on the average number and frequency of the total mutations, it did affect the spectra of DNA mutations. Compared to CK, the uniform M33 reduced the number of nucleotide transitions while the gradient M24.5 increased amounts of the InDels (> 3 bp). Thus, our data reveals that the strength and gradient can function in an antagonistic or independent manner under the condition of UHSMF. In summary, our evidence supports that exposure of UHSMF has an impact on both physiological and genetic processes, which provides a preliminary safety assessment for the usage of smart phytoprotection facilities in the future.

In spontaneous mutations, the rates of base substitutions and InDels were estimated to be around 7 × 10^-9^ and 1.3 × 10^-9^ per site per generation, respectively (Ossowski et al., 2010). However, our estimated rates of SNVs and InDels in CK (Col-0) were 9.55 × 10^-9^ and 0.89 × 10^-9^ per site per generation, respectively. We assume that the higher mutation rates of base substitution could be attributed to the following reasons. One is the coverage depth of genomic resequencing and the length of reads. It is generally accepted that the more reads produced from the next-generation sequencing, the more opportunities to call mutations. In addition, genome coverage can be significantly improved with longer read lengths (Chan, 2009). On average, our sequencing depth (48.56×) for each plant and the length for each read (150 bp) were about 2.6- and 3.7-folds of those reported by Ossowski et al. (2010), respectively, which might result in more mutations discovered in our study. Second is the different analytic softwares used to call mutations. Numerous software differing in accuracy and efficiency have been developed to map short reads to the reference genome and to detect various variants (Schilbert et al., 2020). In addition, recent reports have indicated that based on next generation sequencing platforms InDels are often severely under-estimated due to difficulties in accurate InDel detection (Wala et al., 2018). The combined tools we used for read mapping and variant calling were Bowtie2 and GATK3, respectively, whose performance were recently evaluated to be of high specificity but relatively low sensitivity by comparison with other combinations, such as BWA-MEM and GATK (Schilbert et al., 2020). Third is the presence of some false positive mutations that account for 3.57% of the total estimated SNVs and were not removed in the estimated rate. Nevertheless, a number of common features of genomic mutations, such as the prevalence of SNVs over InDels, higher frequency in DNA transitions than transversions, and higher ratio of deletions to insertions in InDels, were also observed in the study. Thus, the results obtained from the data analytic pipeline we used are reliable and consistent with previously reported.

It is well known that electromagnetic waves with high frequency such as X-rays and gamma rays can induce DNA damages including strand-breaks and base modification (Basu, 2018). Recently, extremely low frequency magnetic fields (ELF-MFs) were also shown to reduce DNA and chromosome stability (Yokus et al., 2008; Elhiti et al., 2018), and probably to cause childhood leukemia (Schüz, 2011). Although non-ionizing radiation of SMF (0 Hz) is thought to be safe for DNA, several experimental results have been demonstrated that UHSMF has a potential effect on DNA stability or mutations (Nakahara et al., 2002; Zhang et al., 2003). Here, genome-wide examination revealed that UHSMF exposure can alter the spectra of mutations including transition and large InDels but not the rate of mutations in *Arabidopsis*. Recently, it was reported that the spontaneous mutation rate is maintained at a stable level even in a minimal cell (Moger-Reischer et al., 2023), which is distinctively different from the mutation rate caused by physical mutagens like gamma rays (Du et al., 2022). Therefore, we speculate that UHSMF does not directly but rather indirectly affect genomic stability, via such as DNA repair process.

The decreased proportion of transitions in SNVs under UHSMF is similar to those induced by irradiation of carbon-ion beam, fast neutron and gamma rays (Belfield et al., 2012; Du et al., 2017; Du et al., 2022), but differs from those induced by regeneration and chemical mutagenesis where the G:C → A:T transition rates increased (Martín et al., 2009; Jiang et al., 2011), and environmental stresses (heat, warming and salt) (Jiang et al., 2014; Lu et al., 2021). It has been indicated that the molecular mechanisms underlying the high rate of G:C → A:T transitions are largely associated with the combined effect of deamination of methylated cytosines (Coulondre et al., 1978; Duncan and Miller, 1980) and dipyrimidine dimers induced by ultraviolet light (Friedberg, 2003; Ikehata and Ono, 2011). In the present study, we found that the highest probability of the base adjacent to the mutated C was T in both CK and UHSMF groups, and the total percentage of T and C adjacent to the mutated C was almost the same among all the groups (**Supplementary Figure 5**). Thus, we assume that dipyrimidine dimers induced by ultraviolet light may not be involved in UHSMF effect on base transition. However, we found that the upstream flanking sequences (-3 to -1) of G and C are AAT and TTA, respectively, which are correlated to the decreased transition rate in UHSMF groups (**Figure 3C**). It will be an interesting question whether these flanking sequences are indeed associated with the G:C → A:T transition rates in the future.

Another mutational spectrum altered by UHSMF is the increased number of InDels larger than 3 bp. Among these larger InDels, we found that homopolymers and polynucleotide repeats are detected largely in M30.5 and M24.5 samples, respectively. Since InDels are readily to form through replication slippage at or close to homopolymer and polynucleotide repeat regions (Viguera et al., 2001), it seems that gradient UHSMF can activate the process of DNA replication slippage (Bennett et al., 2020). In addition, InDels can be generated through various DNA repair pathways that are activated by DNA damages including mismatch bases, DNA single- and double-strand breaks, and intra- and inter-strand cross-links (Tuteja et al., 2001). The size of InDels is closely associated with mechanisms of DNA repair. For example, among the four major double-strand breaks (DSB) repairing pathways, namely homologous recombination (HR), non-homologous end joining (NHEJ), microhomology-mediated end joining (MMEJ) and single-strand annealing (SSA), NHEJ is the dominant pathway because of its active in most cell cycle phases and can perfectly repair DSB as HR or produce small InDels with a few bases in size, while MMEJ is mainly active in S and G2 phases and leads to InDels that are larger than NHEJ InDels but smaller than 30 bp. InDels caused by SSA are minor because the frequency of a long homology stretch in the vicinity of DBS is much smaller than that of a microhomology. Based on our evidence showing that the number of the InDels being larger than 3 but smaller than 30 bp increase with the increase of the gradient level, we assume that MMEJ might be the major DSB repair pathway under UHSMF.

To date, lots of reports have been demonstrated that treatment of middle or weak strength SMF can increase seed germination (Zhang et al., 2017b; Sarraf et al., 2020). Consistently, we also found that the uniform M33 promoted seed germination despite of not significantly different from the CK. In contrast, our data indicated that the rate of seed germination gradually decreased with the increase of magnetic gradient. The 150 T/m gradient in M24.5 significantly inhibited seed germination, compared to the 95 T/m gradient in M30.5 and the uniform field in CK and M33. Thus, the effect of UHSMF on seed germination depends on both strength and gradient. Since seed germination is an important agronomy trait that significantly affects crop growth and resistance to biotic and abiotic stresses, utilization of uniform SMF may provide an efficient presowing seed treatment for sustainable agriculture. It is important to elucidate molecular mechanisms by which SMF promotes or inhibits seed germination in the future.

In conclusion, our findings suggest that short time exposure of *Arabidopsis* dry seeds to UHSMF with maximum intensity of 33.0 T or gradient of 150 T/m should be safe for plant genomes. However, UHSMF exposure can alter the spectra of DNA mutations, which is correlated to the intensity and gradient. In the future, an attention should be paid to both SMF strength and gradient in developing smart phytoprotection with UHSMF, which is a promising research field but faces important challenges in elucidating molecular mechanisms for the adverse magnetobiological effects on plant growth and development.

## Materials and methods

### Exposure system and treatment of UHSMF

UHSMF up to 33.0 T used in this study were produced by a WM5 water-cooled resistive magnet (Gao et al., 2016; Tian et al., 2021) (High Magnetic Field Laboratory of Chinese Academy of Sciences). The temperature inside the device was maintained at 22-24°C by utilizing thermal conduction from temperature-controlled water that circulated through the gap between the inner tube and outer tube. Dried seeds from a single plant (Col-0) were set in five layers with each 50 mm working bore space. The UHSMF parameters for each layer has been summarized in **Figure 1A**. The UHSMF exposure were lasted for 1 h.

### Plant sampling and DNA sequencing

After UHSMF exposure, the seeds were surface-sterilized and stratified at 4°C in dark for 2 days, and then sown on the half-strength Murashige and Skoog (MS) medium (pH 5.7) (Sigma-Aldrich) containing 0.7% (w/v) phyto agar and 1% (w/v) sucrose. Seeds were germinated at 20°C under 100 μmol·m^–2^·s^–1^ white light with long-day conditions (16 h light/8 h darkness), and at least 50 seedlings for each treatment were transplanted into soil. All the plants were self-fertilized for one generation.

For DNA extraction, rosette leaves were sampled from the second generation plants, and 40 individual plants were used for each treatment. Extracted genome DNA was qualified by checking gel electrophoresis and OD260/280. DNA concentration was accurately quantified by using Qubit. Samples with OD values between 1.8 - 2.0 and DNA content above 1.5 µg were used for library construction. Qualified DNA samples were randomly broken into 350 bp fragments and then constructed by TruSeq Library Construction Kit. Sequencing was done by Illumina platform paired-end 2 × 150 bp sequencing lane. Raw reads in FASTQ format were gotten for the further analysis.

### Variant calling and genotyping

Totally 236 lines were generated for whole-genome sequencing, with five treatment groups and one control group. Raw reads from sequencing were mapped onto the reference genome (TAIR10) using Bowtie2 (Langmead and Salzberg, 2012) software (version 2.3.2) after quality control. Samtools (Li et al., 2009) (version 1.5) was used to convert the mapped sam files to bam files. Then, potential PCR duplicates were removed by using “MarkDuplicates” in picard (version 1.119, https://broadinstitute.github.io/picard/). We employed GenomeAnalysisTK (McKenna et al., 2010) (version 3.4-0) with “RealignerTargetCreator” to identify and generate a list of target intervals. Furthermore, “InDelRealigner” was utilized to realign alignments around small InDels. “UnifiedGenotyper” in GATK was applied to call raw variants. The parameter for the variants calling was “-stand_call_conf 30, -stand_emit_conf 10”.

### SNV filter and annotation

To reduce the error rate of variant calls, the filtering threshold for the variants are shown below: removal of loci with deletions greater than 20% and heterozygosity greater than 20%. For the loci that are reserved for variation in only one material, we call them unique site. SNPeffect (Cingolani et al., 2012) (version 3.6b) with parameter “-no-intergenic -no-downstream -no-upstream -no-intron -no-utr” to predict the molecular and structural effects of protein-coding variants. SIFT4G (Vaser et al., 2016) was used to predict whether an amino acid change would affect the function of a protein.

### Variant confirmation via sanger sequencing

The backup of harvested rosette leaves which stored in -80°C were used for variant confirmation. DNA of leaves were extracted by TPS buffer (1M KCl, 0.1M Tris-HCl, 0.01M EDTA-2Na). The candidate sites were randomly selected and specific primers were designed (**Supplementary Table 4**). For each selected site, the samples containing variant and not (at least 3 lines) were both checked. DNA fragments containing candidate sites in the center with at least 400 bp were cloned from genomic DNA and confirmed by sanger sequencing. The reads were aligned with candidate reference sequences and the sequencing peak diagrams were checked.

### Seed germination assays

The surface-sterilized seeds were stratified at 4°C in dark for 2 days and then sown on the half-strength Murashige and Skoog (MS) medium (pH 5.7) (Sigma-Aldrich) containing 0.7% (w/v) phyto agar and 1% (w/v) sucrose. Seeds were germinated at 20°C under 100 μmol·m^–2^·s^–1^ white light with long-day conditions (16 h light/8 h darkness). The seeds of SMF-treated and control were sown on the same plate and counted at 24, 48 and 72 h after sowing. The statistical analysis was performed by one-way ANOVA with Tukey`s multiple comparison test and statistically difference was confirmed by *P* < 0.05.

## Data availability statement

The data presented in the study are deposited in the European Nucleotide Archive (https://www.ebi.ac.uk/ena/browser/home), accession number PRJEB65433.

## Author contributions

XX: Formal analysis, Visualization, Writing – original draft. MC: Data curation, Formal analysis, Writing – original draft. TC: Writing – original draft. XN: Writing – original draft. ZF: Conceptualization, Writing – review & editing. YF: Conceptualization, Writing – review & editing. LZ: Writing – review & editing, Resources. XZ: Writing – review & editing, Resources. JH: Conceptualization, Writing – review & editing, Methodology.

## Supplementary material

Supplementary Table 1 List of variants.

Supplementary Table 2 InDels flanking sequences.

Supplementary Table 3 Metascape result.

Supplementary Table 4 Primers used for conformation.
