# Supplementary material for: Ultra-high static magnetic field induces a change in the spectrum but not frequency of DNA spontaneous mutations in Arabidopsis thaliana
Source: Front Plant Sci. 2023 Dec 6;14:1305069. doi: 10.3389/fpls.2023.1305069 (PMC10731980; doi:10.3389/fpls.2023.1305069)
Supplement: Supplementary file 5 [file DataSheet_1.docx]

**
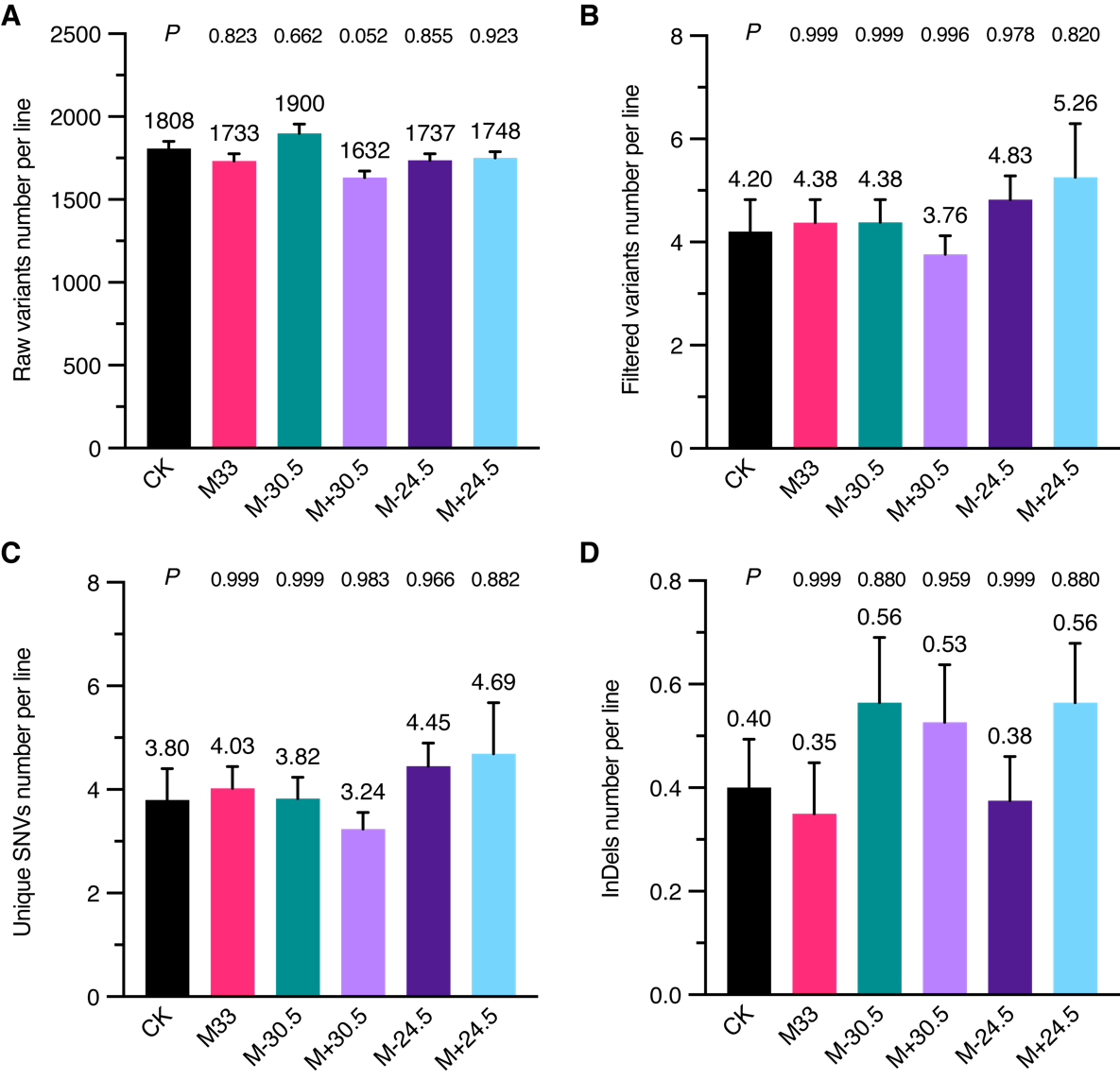
Supplementary Figure 1 Comparison of variants between CK and five SMF-treated groups.**

**A-D** Average number of raw variants (A) and filtered variants (B) including unique SNVs (C) and InDels (D). Mean of each group and *P* value in a-d are noted. *P* represents the difference between noted group and CK (one-way ANOVA Tukey`s multiple comparison test). Error bars represent standard error of the mean.

**
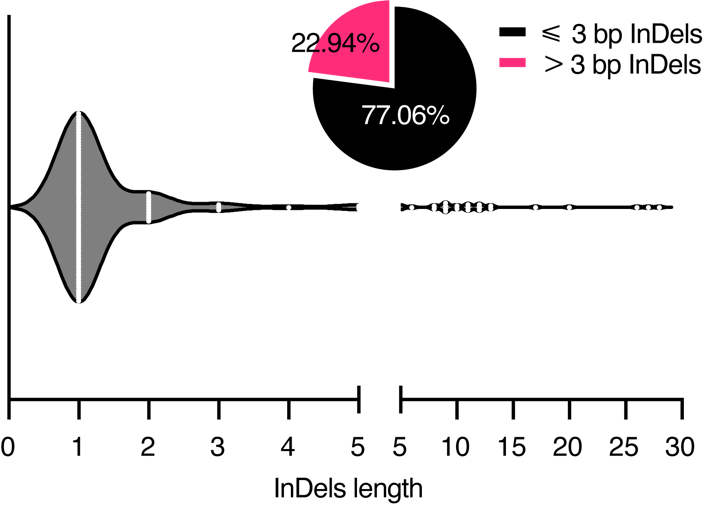
Supplementary Figure 2 Composition and distribution of InDels length.**

The pie chart in the upper right shows the composition of InDels based on their length with 3 bp cutoff. The violin plot with all data in white dots shows the distribution of InDels length.

**
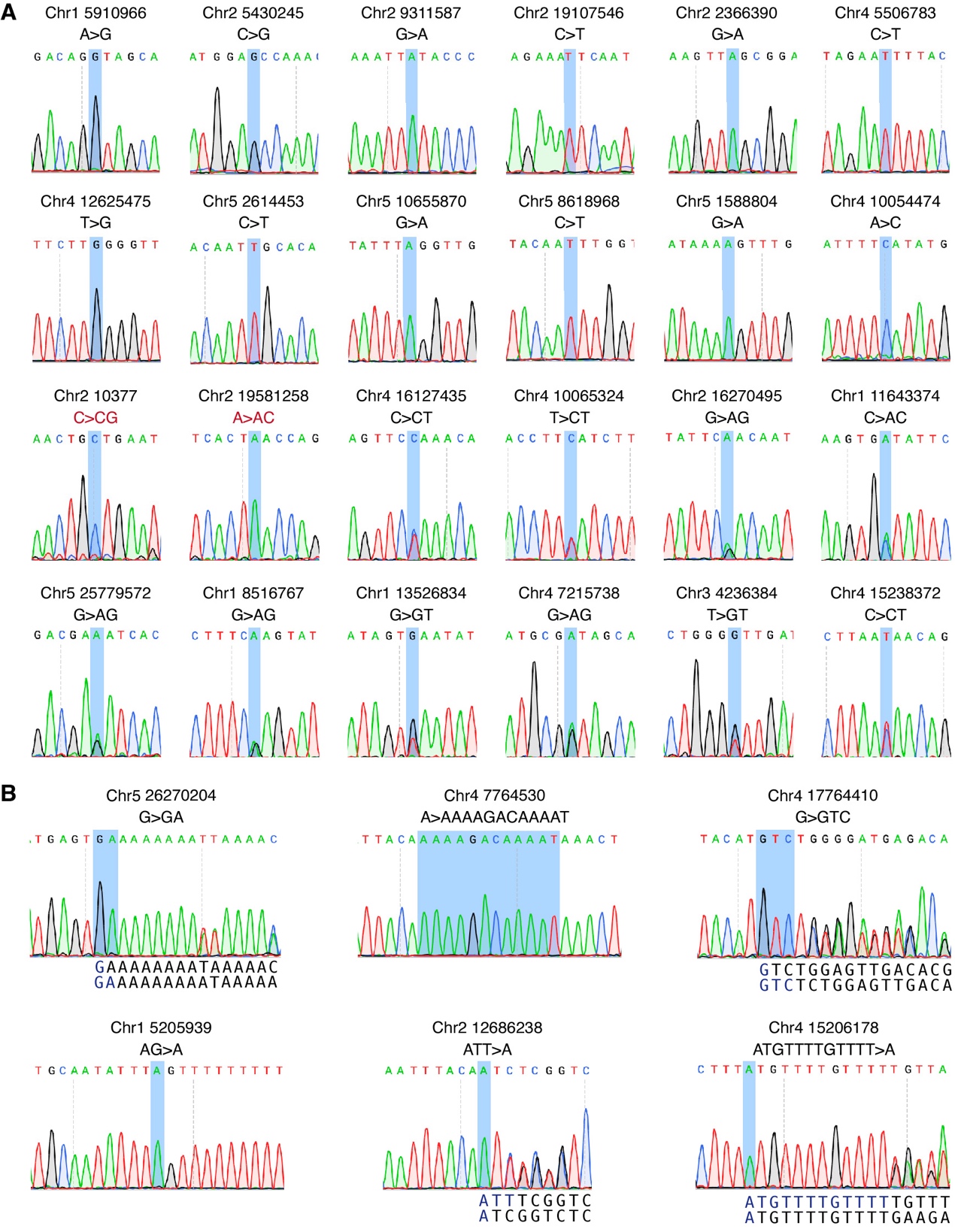
Supplementary Figure 3 The representative sequencing peak diagrams for conformation of the unique SNVs and InDels.**

The unique SNV and InDel located site is noted in the top of the diagrams and marked by blue rectangle. Chr means chromosome. **A** The base substitution results from the high-throughput sequencing are showed in the up of the diagrams and the letters in red mean the Sanger sequencing results are not in accordance with the high-throughput sequencing. Two base letters together indicate the heterozygosis. **B** InDels results from the high-throughput sequencing are showed in the up of the diagrams. For heterozygosis, sequences are noted below the diagrams.

**
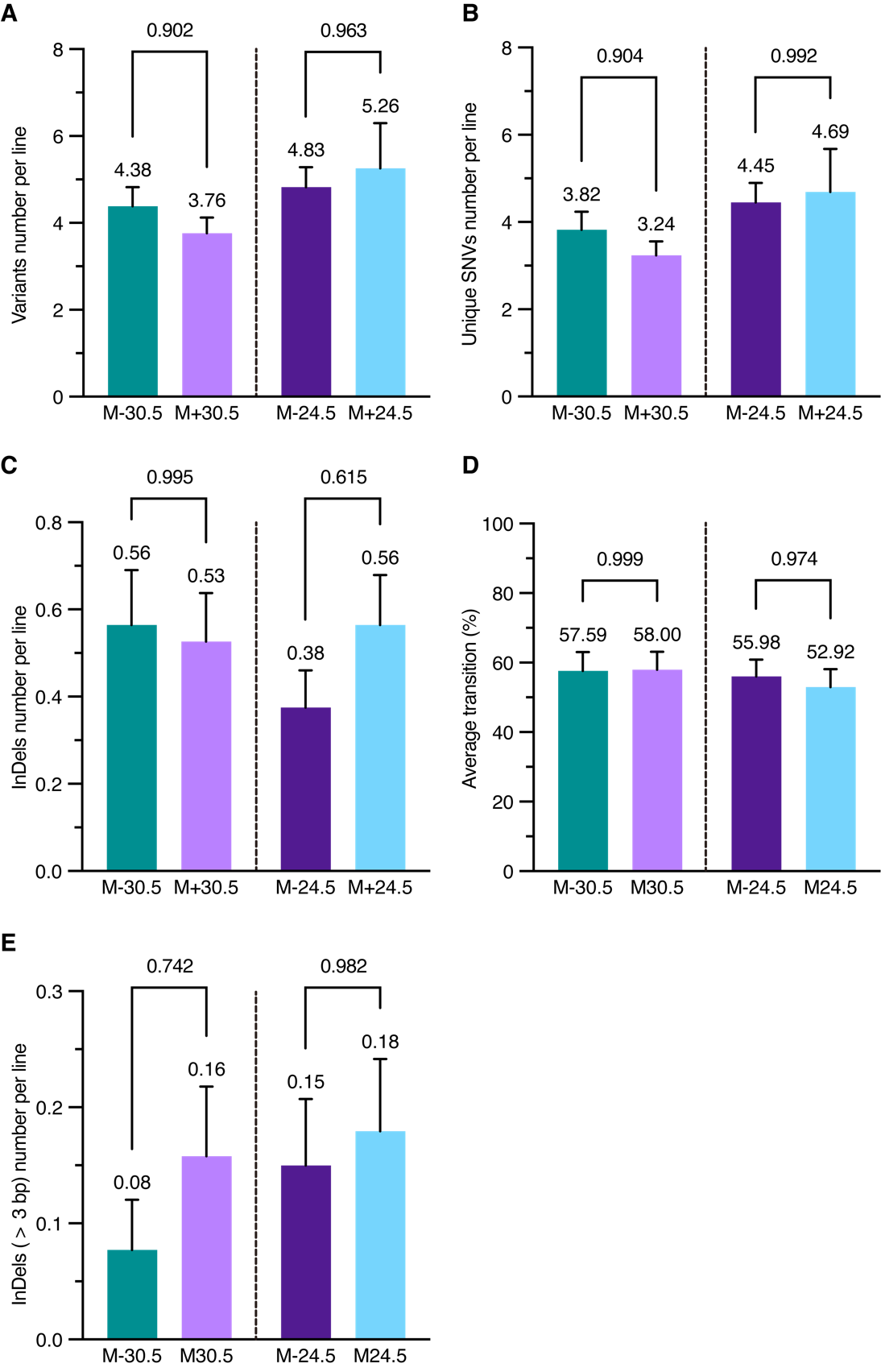
Supplementary Figure 4 Comparison of variants in SMF-treated groups with opposite SMF gradient direction.**

**A-C** Average number of filtered variants (A) including unique SNVs (B) and InDels (C) in M-30.5, M+30.5, M-24.5 and M24.5. **D** Average percentage of base transition in M-30.5, M+30.5, M-24.5 and M24.5. **E** Number of InDels larger than 3 bp per line in M-30.5, M+30.5, M-24.5 and M24.5. Mean of each group and *P* value are noted. *P* represents the difference between noted groups (one-way ANOVA Tukey`s multiple comparison test). Error bars represent standard error of the mean.

**
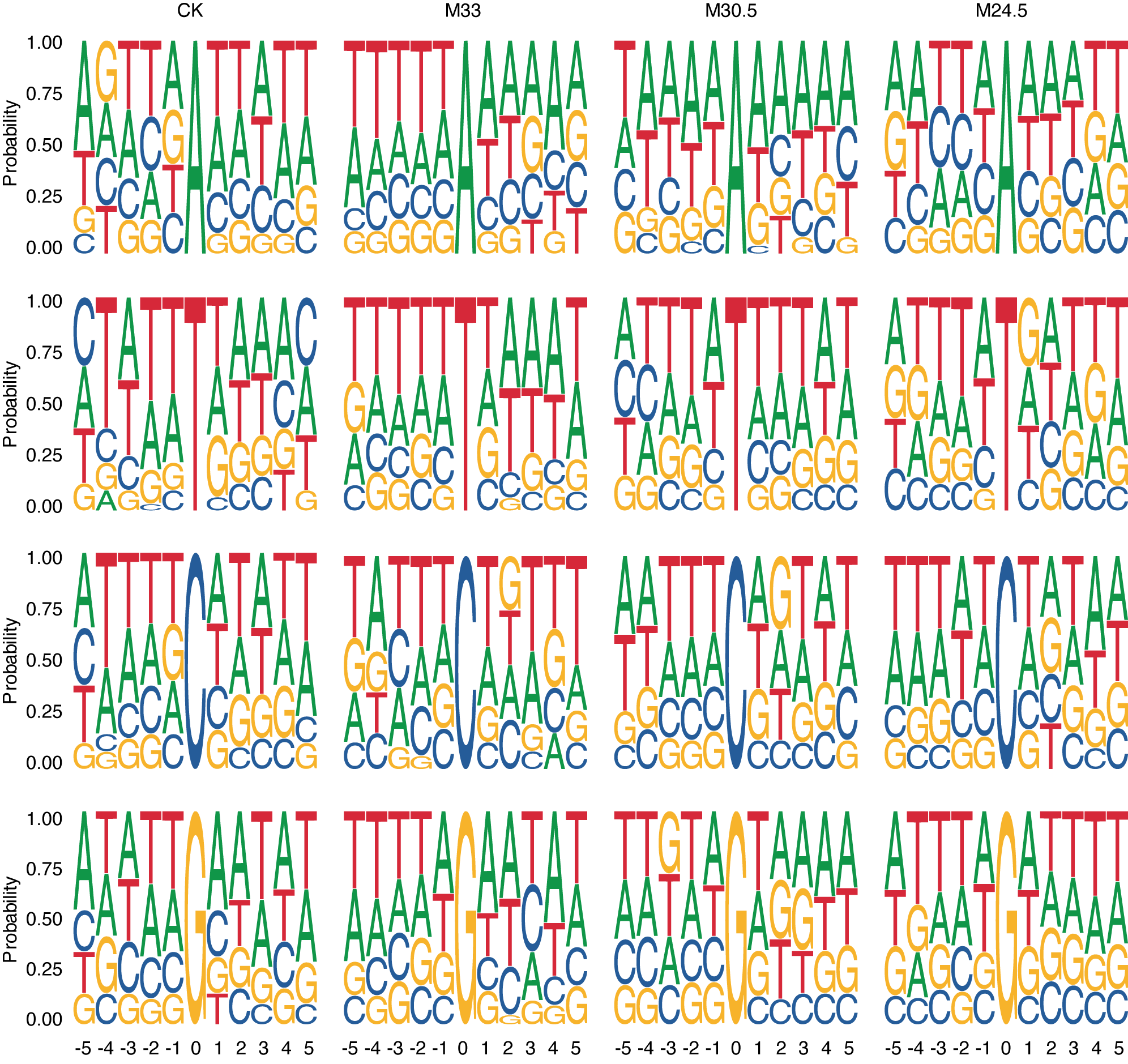
Supplementary Figure 5 Motif analysis for the unique SNVs.**

Unique SNVs with A, T, C and G are divided into four groups and visualized respectively. With up and down stream 5 nucleotides, the unique SNVs are located in the middle of motif which is 0. The height of base letters shows their proportion in the corresponding site.

**
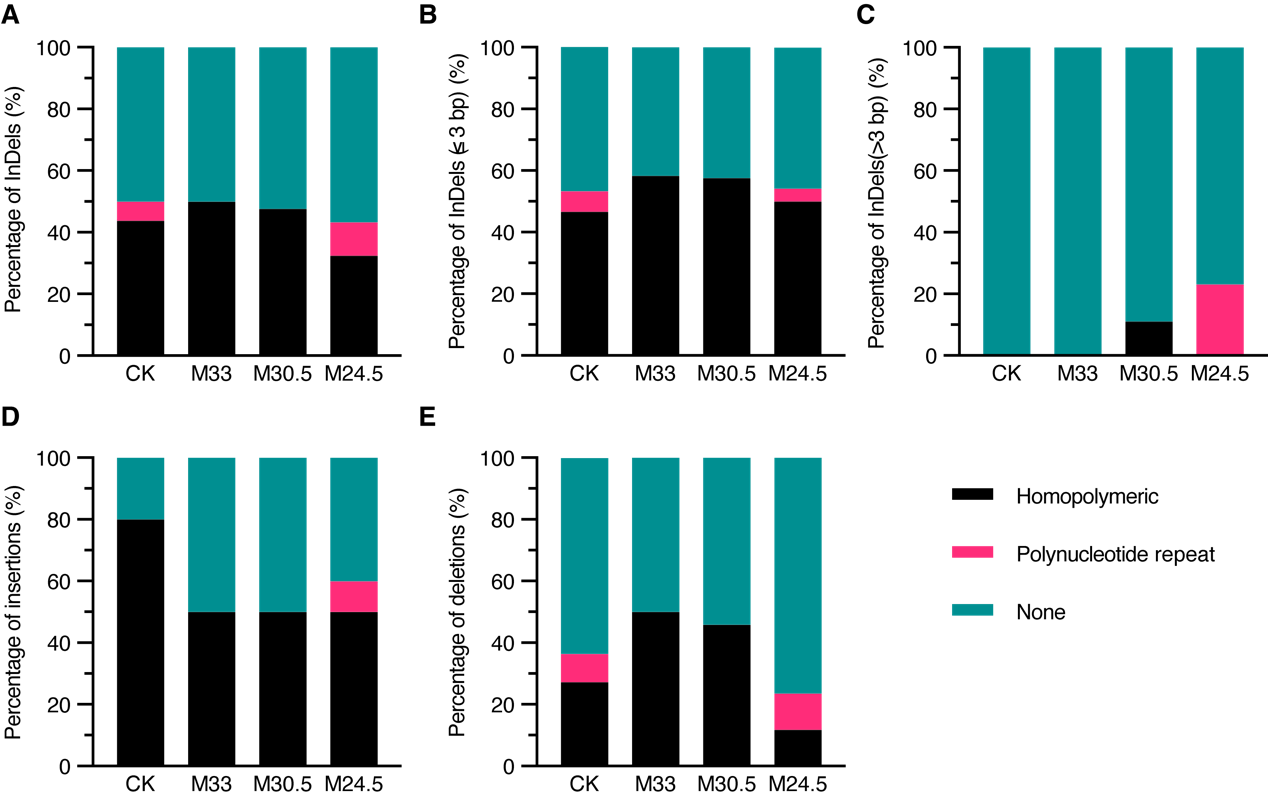
Supplementary Figure 6 Comparation of InDels flanking sequences.**

**A** Characteristics of InDels flanking sequences among CK and UHSMF treated groups. **B, C** The comparation for flanking sequences of the InDels ≤3 bp (B) or >3 bp (C). **D, E** The comparation for flanking sequences of insertions (D) and deletions (E).


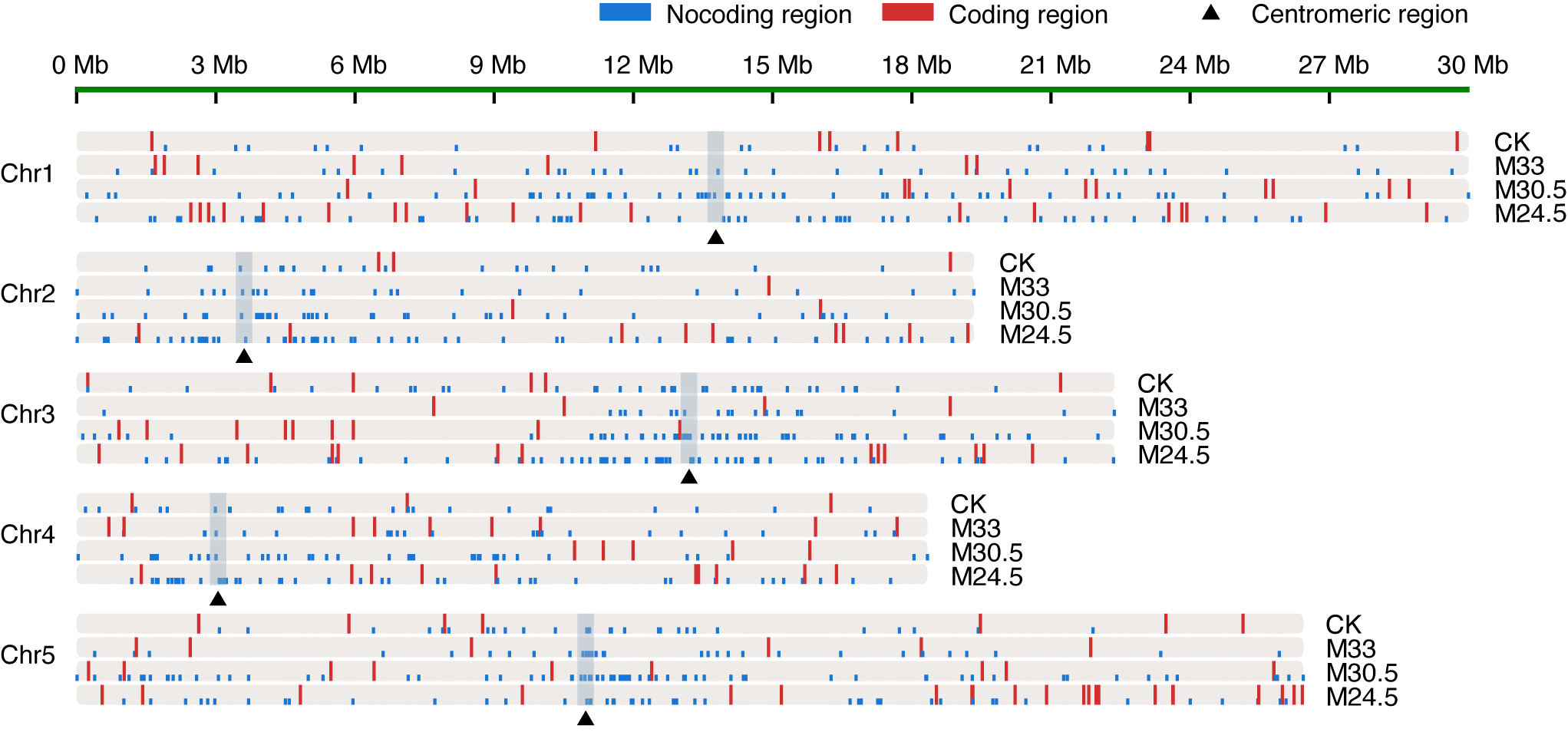
**Supplementary Figure 7** **Distribution of variants across chromosomes.**

Variants in noncoding and coding regions are marked by blue short lines and red long lines. Centromeric regions are labeled by black triangle. Mb, megabase.
